# Impact of Excessive Noise Generation in Orthopaedic Operating Theatres: A Comprehensive Review

**DOI:** 10.7759/cureus.54469

**Published:** 2024-02-19

**Authors:** Madhan Jeyaraman, Naveen Jeyaraman, Sankalp Yadav, Arulkumar Nallakumarasamy, Karthikeyan P Iyengar, Vijay Jain

**Affiliations:** 1 Orthopaedics, Viriginia Tech India, Dr. M.G.R. Educational and Research Institute, Chennai, IND; 2 Orthopaedics, ACS Medical College and Hospital, Dr. M.G.R. Educational and Research Institute, Chennai, IND; 3 Medicine, Shri Madan Lal Khurana Chest Clinic, New Delhi, IND; 4 Orthopaedics, Jawaharlal Institute of Postgraduate Medical Education and Research (JIPMER) Karaikal, Karaikal, IND; 5 Orthopaedics and Trauma, Southport and Ormskirk Hospital NHS Trust, Mersey and West Lancashire Teaching NHS Trust, Southport, GBR; 6 Orthopaedics, Atal Bihari Vajpayee Institute of Medical Sciences, Dr. Ram Manohar Lohia Hospital, New Delhi, IND

**Keywords:** operation theatres, hearing loss, nihl, noise, orthopaedics, noise-induced hearing loss (nihl)

## Abstract

Excessive noise in the orthopaedic operating theatre (OT) is an underrecognized and often neglected health hazard noticed amongst surgeons, patients and theatre and scrub practitioners. A comprehensive search strategy was conducted using databases, such as PubMed, Scopus and Web of Science, with the search words 'noise', 'NIHL' and 'orthopaedics' to retrieve the significant data and generate this narrative review. We evaluated the typical causes, potential hazards and negative effects of noise-induced impacts on OT personnel and patients. Strategies to mitigate the effects of unnecessary, disproportionate noises in the OT environment were explored. Excessive noise generated in orthopaedic OTs can produce several negative effects on patients, surgeons and staff. Noise-induced hearing loss (NIHL) is a rare and under-noticed disorder. The orthopaedic OT environment, with the ever-increasing use of power tools and surgical instruments, contributes to detrimental noise generation. NIHL is an occupational hazard. Raising awareness, appropriate training and clinical governance in collaboration with the hospital risk management team amongst all the medical and paramedical fraternities working in orthopaedic theatres can mitigate challenges faced due to the deleterious effects of excessive noise. We propose recommendations and standard operating protocols that can be incorporated into hospital policies to prevent NIHL among the orthopaedic fraternity and patients alike.

## Introduction and background

Occupational noise exposure of an excessive nature can have a deleterious effect on workers, healthcare professionals, staff and patients in the health industry [1,2]. The orthopaedic operating theatre (OT) environment with excessive noise generated due to power tools, such as oscillating drills and machines, can lead to communication difficulties, hearing damage and, more worryingly, impaired cognitive performance, leading to surgical errors in the care of the patient [3]. Treating orthopaedic surgeons can experience a multitude of aural and extra-aural effects. Hazard effects, such as noise-induced hearing loss (NIHL) and other extra-aural impacts, may have various systemic and psychological implications [4].

Power tools, plaster-cutting saws, bone-cutting saws, suction and the striking of hammers can generate noise as loud as 145 dB [5]. Hearing loss due to noise exposure could sometimes be permanent. The gradual hearing loss due to prolonged noise exposure at the workplace is well known as NIHL [6,7]. The available literature depicts that half of the personnel working in orthopaedic theatres are at a greater risk of suffering from varying grades of NIHL [4]. The previous work starkly highlights how orthopaedic surgeons are at a high risk of stress, infection, exposure to radiation, chemicals and musculoskeletal problems, but there is very little focus on noise-related hazards, which are indeed encountered regularly when performing surgeries using manual and mechanized tools [8].

The identified plausible contributing factors causing hearing impairment include exposure to steady noises for longer durations and impulsive noises for shorter durations. The hospital zone is categorized as a silent zone. However, noise generation is seemingly inevitable. As per the noise pollution (regulation and control) rules (2000) in India, silence zones, which include hospitals as one of the setups, are permitted to have a noise level of only 50 dB (A) leq during the day and 40 dB (A) leq at night [9,10]. The permissible limit in the operation theatre setup in our nation is not aptly defined. As per the National Institute for Occupational Safety and Health (NIOSH), workers who are exposed to an average noise level of 85 dBA daily over a 40-year working span have an 8% higher risk of material hearing impairment. Moreover, the risk increases by 25% if there is an average daily noise exposure of 90 dBA [11,12].

**Figure 1:**
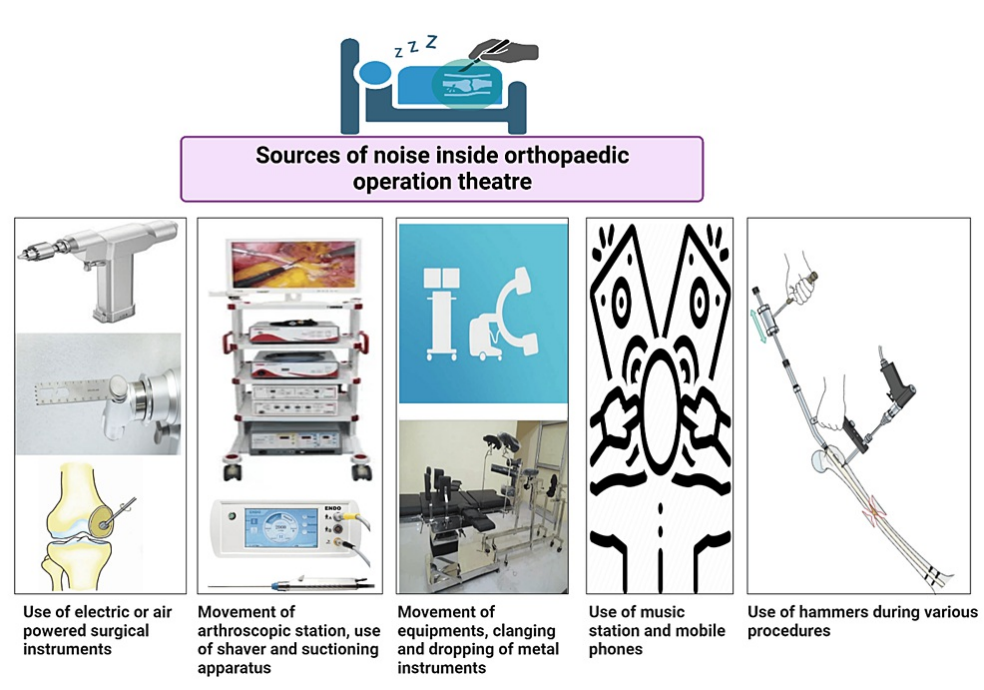
Sources of noise inside an orthopaedic operation theatre Picture courtesy: Dr. Madhan Jeyaraman

Orthopaedic surgery usually involves procedures like joint replacement, which utilizes mechanized saws and drills for preparing and placing bones in appropriate alignment in order to fix a new prosthesis. The employed tools for the same are classically high-powered devices that permit surgeons to precisely make dimensional cuts as best suited to accept the newer prosthesis. Notably, powered instruments used during orthopaedic surgery serve as the primary source for noise generation in operation theatres and thereby call for expediting mitigation strategies to prevent NIHL. Furthermore, hammering in trauma during intramedullary nail insertion and arthroplasty implants, cutting osteotomy sites through osteotomes and burring of bony cavities, among others, can generate lots of noise, which could be detrimental to orthopaedic surgeons. This detailed review aims at addressing a significant health issue that is often neglected by surgeons and may have a detrimental effect on surgeons and paramedical staff working in the operation theatre. The sources of noise inside an orthopaedic operation theatre are depicted in Figure 1. 

## Review

Evidence based on the literature

Arthroplasty surgeons have a significantly greater prevalence of hearing loss than their non-surgical counterparts, according to the Hearing Evaluation of ARthroplasty Surgeons (HEARS) study [13]. The orthopaedic staff is highly on the verge of getting exposed to intense noise levels for longer periods, which is liable to cause NIHL. We measure noise logarithmically in decibels (dB), such that an increment of 3 dB is equal to doubling the noise dose. It is to be noted that exposure to 90 dB for eight hours is the same as exposure to 105 dB for 15 minutes. It has been found that intense exposure to noise causes damage to the outer hair cells within the cochlea. The stereocilia of the outer hair cells become more flexible, which results in a poor response to stimulation. As the intensity and duration of exposure increase, the severity of damage is equivalently reciprocated due to fusion and loss of stereocilia. This causes the loss of outer hair cells and the formation of scar tissue. Moreover, continuous exposure might also damage the inner hair cells and supporting cells in the organ of Corti, along with secondary neural degeneration [14]. The otoacoustic emissions render a quick, objective insight into cochlear damage prior to any change in pure tone audiometric thresholds [15]. It is sensitive in detecting differences in the hearing threshold, making it ideal for monitoring high-risk staff for hearing loss due to noise exposure.

Orthopaedic operation theatres are too noisy as they involve the use of oscillating saws, impacting components with a hammer, reaming, rasping and others, such as cautery tone, surgical suctions and fans in space suits, while conducting surgical interventions [16]. The literature is enormous on orthopaedics and surgical aspects; however, only a handful is available to shed light on the self-risk incurred by the operating team. Willett disclosed that 22 senior orthopaedic personnel suffered from NIHL due to the frequent usage of air-powered and electric drills and saws [17]. Siverdeen et al. showed that the noise levels in orthopaedic theatres generated by the usage of tools can be hazardous to members of staff and patients [18].

Mullet et al. tested a variety of instruments used routinely in both elective and trauma surgery and showed that all instruments generated noise levels greater than the threshold for routine monitoring of hearing loss under health and safety legislation [19]. These include the compact air drill, Desoutter drill, plaster saw, oscillating saw, reciprocating saw, AO drill, MicroAire drill, hammering and disconnecting air hose, which were all evaluated at sound pressures of 5 cm, 15 cm, 1 m and 2 m. The maximum sound produced was with the disconnecting air hose and the least with the AO drill, respectively. Modern instruments nowadays tend to generate less noise, but this does not pardon the associated hazards.

Peter et al. measured noise levels during total knee arthroplasty during cutting and hammering, wherein they compared noise production with a conventional sagittal oscillating blade system with a full oscillating blade and two newer oscillating tip saw systems (handpiece and blade). The findings of the study suggest that the level of noise produced was significantly lower for the oscillating tip saw systems when compared with the conventional saw system; however, all came within the range causing NIHL. Moreover, the conventional system handpiece generated a significantly higher noise level in comparison to the oscillating tip handpiece [20]. These findings have well-enunciated higher noise levels in orthopaedic OTs, which were greater than 110 dB [21]. While the presence of music in OTs contributes to overall noise levels, research suggests that it may potentially reduce anxiety and stress levels among individuals involved in surgical procedures [22].

Health hazards due to noise exposure

NIHL is an under-reported and silent health hazard among healthcare personnel in orthopaedic theatres. Such a silent health hazard has two categories of effects in HCPs, namely, aural effects, such as NIHL, and extra-aural effects, such as stress, anxiety, psychological symptoms and cardiovascular effects.

Such aural effects are determined by the amount of noise exposure in orthopaedic OTs and can set a safe level of workplace noise exposure in their daily healthcare work. In this regard, the United Kingdom declared noise exposure under 'The Control of Noise at Work Regulations 2005' to consider a) 'steady' noise for long-term exposure and b) impulsive noises for short-term exposure. The Occupational Safety and Health Administration (OSHA) declared that the maximum exposure to noise must be within 90 dB for eight hours in a day, whereas the NIOSH reported 85 dB for eight hours in a day [23,24].

The powered instruments used during primary replacement surgeries, orthopaedic oncological surgeries and complex trauma surgeries generate a considerable amount of noise in the orthopaedic OT. Such noise induces a risk of developing NIHL among the healthcare providers (HCPs) present in the OT and its complex. Various studies have documented minor hearing loss in 50% of HCPs with five to 22 years of noise exposure in orthopaedic OTs [25,26]. Siegel opined on recommending the inclusion of orthopaedic surgeons in the prevention of hearing loss program because the noise generated in OT exceeded the noise occupational exposure limits (104 dBA with levels of 85 dBA in 10-18% of the time) [25].

Fritsch et al. reported the highest noise level of 131 dB with the spectral recordings of 20 major surgical instruments in orthopaedic OT [27]. Kamal reported that 50% of HCP in orthopaedic OTs suffer NIHL other than infections, radiation or chemical exposures and musculoskeletal hazards [28]. HCPs experience noise and vibrations while using manual orthopaedic instruments [8]. Kwan et al. reported that microdiscectomy generated maximum noise of more than 11.3% of the maximum allowable daily dose and 104.1% of the maximum projected daily dose. According to the authors, orthopaedic surgeons frequently encounter harmful noise levels (i.e. >85 dB), which puts them at risk for long-term hearing damage [29].

Love reported the maximum allowable noise exposure during total hip replacement to be within limits (average of 4.5%; ranging from 1.52% to 6.45%) and knee (average of 5.74%; ranging from 4.09% to 7.39%), and transient peak noise of 140 dB was observed on multiple surgical sessions [3].

Noise levels vary with every procedure in orthopaedic OT (cast removal: 76 dB for eight hours/day; total hip/knee replacement: 105.6 dB with mallet usage or 97.9 dB with oscillating saw usage; high-speed gas turbine drills: 118 dB; and suction tips with trapped tissues: 96 dB for 8 hours/day) [30-32]. Hence, the noise generated in orthopaedic OTs exceeds the safety level set by the OSHA and NIOSH.

There are no international standards for safeguarding orthopaedic surgeons and OT staff against noise exposure. Regardless of the duration of exposure, hearing protection is advised for values >85 dB. HCPs in orthopaedic OTs are recommended to undergo a hearing conservation program and regular audiometric testing and adapt to wearing hearing protection aids, such as flat attenuation earplugs or earmuffs with electronic noise cancellation features. Wearing such noise-reducing aids should not interfere with communication among HCPs in OTs. Westone TRU custom hearing protection with noise reduction rating (NRR) of 10 and 15 filters is effective for noise reduction [33].

Recommendations

Conducting an induction program to raise awareness about noise-associated health hazards at the workplace. All medical and paramedical personnel working in operation theatres should be mandated to attend in order to learn preventive measures. Periodic formal assessment of noise production in the OT should be done using appropriate measuring devices and modifying preventive strategies. Orthopaedic surgeons should actively participate in devising standards of protocol to be followed on OT premises in order to defer this identified health hazard.

Specialized devices, including high-fidelity earplugs, can be used while performing surgical procedures, generating noise without breaking the sterilized field of surgery, and shall render a practical solution to this issue. Hospital-based screening programs for health personnel at risk should be formulated with the purpose of the earliest identification of NIHL.

Orthopaedic society shall maintain a database with such events and further direct course of action to minimize them. Personalized efforts include inculcating the habit of practicing relaxing yoga techniques, meditation, and going to quieter places as and when permissible.

The proposed standard operating protocol for reducing NIHL in orthopaedic theatre personnel is tabulated in Table 1.

**Table 1:** Proposed standard operating procedure (SOP) for reducing NIHL in orthopaedic theatre personnel SOP: standard operating procedure, OT: orthopaedic theatre, HCP: healthcare personnel, NIHL: noise-induced hearing loss

Phases	Significance
I: Pre-employment check-up	Assessment of hearing using available preliminary screening procedures
II: SOPs for operating theatres	Identify high-risk health personnel; All health personnel, especially those at high risk, should strictly adopt healthy hearing prevention practices involving the usage of high-fidelity earplugs. Switch to operating tools that generate less noise without compromising quality; evaluate daily noise production in the operation theatre with an appropriate, cost-effective device; maintain and update the database for daily noise production in the operation theatre.
III: Annual evaluation of orthopaedic OT HCPs	Earliest identification of NIHL: Formulate a specialized task force in the respective hospital setting to address these health hazards and all the associated issues.
IV: Surveillance	Report all the identified instances without any hesitation and direct them to appropriate measures.
V: Education	Periodic awareness and conscience to self-direct for healthy hearing practices.

**Figure 2:**
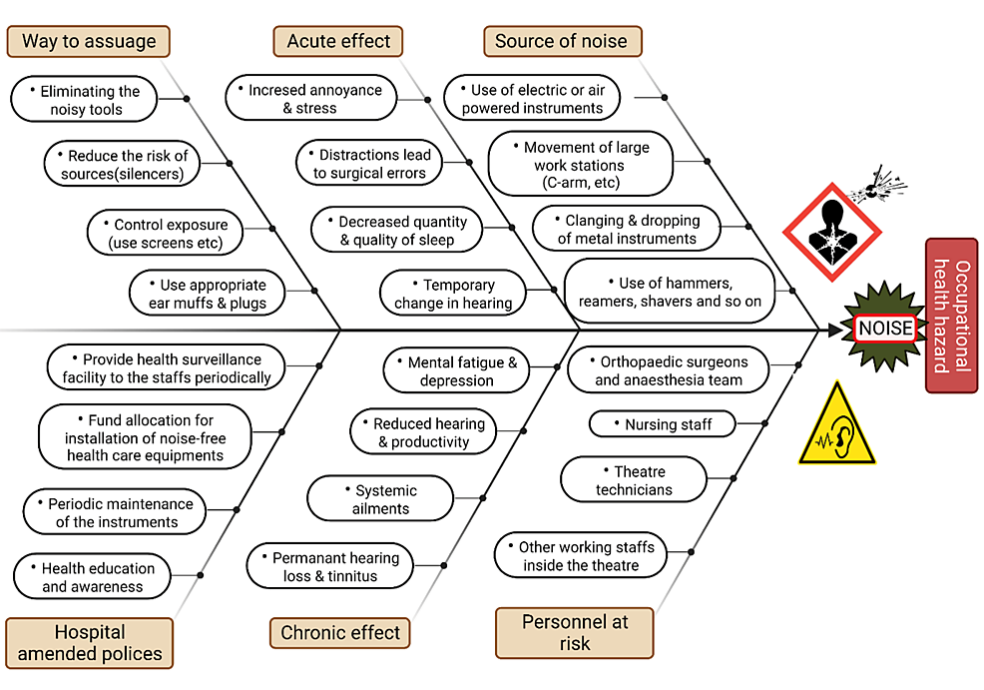
Overview of noise exposure in the orthopaedic operation theatre Picture courtesy: Dr. Madhan Jeyaraman

The overview of the sources of noise, health hazards due to noise exposure and hospital policies to mitigate the noise generated in OTs is depicted in the fishbone representation in Figure 2.

## Conclusions

NIHL is an occupational hazard. Raising awareness, appropriate training and clinical governance in collaboration with the hospital risk management team amongst all the medical and paramedical fraternities working in orthopaedic theatres can mitigate challenges faced due to the deleterious effects of excessive noise. We proposed recommendations and standard operating protocols that can be incorporated into hospital policies to prevent NIHL among the orthopaedic fraternity and patients alike.
